# Flavor evolution in refrigerated tilapia processed by cold plasma: Volatile profiles and odor quality driven by excitation gas-generated reactive species

**DOI:** 10.1016/j.fochx.2026.104118

**Published:** 2026-06-17

**Authors:** Tingting Yang, Xiaohan Sang, Yuanyuan Wang, Xueying Zhang, Guanghua Xia, Liming Zhang, Jiamei Wang, Yaqin Wang, Per Ertbjerg

**Affiliations:** a Hainan Engineering Research Center of Aquatic Resources Efficient Utilization in South China Sea, Key Laboratory of Seafood Processing of Haikou, Key Laboratory of Food Nutrition and Functional Food of Hainan Province, School of Food Science and Engineering, Hainan University, Haikou, Hainan 570228, China; b Department of Food and Nutrition, University of Helsinki, Helsinki FI-00014, Finland

**Keywords:** Cold plasma, Tilapia, Volatile compounds, Lipid oxidation, Flavor modulation

## Abstract

Rapid flavor deterioration due to lipid oxidation and microbial spoilage is a major challenge during refrigerated fish storage. This study was the first to systematically investigate how cold plasma (CP) gas composition (low-oxygen GasA: 10% O_2_; air GasB: 22% O_2_; high-oxygen GasC: 30% O_2_) drives volatile flavor evolution and enzyme-flavor correlations in refrigerated tilapia. A total of 66 volatile compounds were identified, with aldehydes (hexanal, nonanal) and 1-octen-3-ol as key contributors. GasC-CP increased total aldehydes by 64% (from 20.59 ± 0.22 to 33.74 ± 6.20 μg/kg). GasA-CP balanced flavor enhancement with off-flavor suppression (hexanal OAV = 1.59 at day 8). OPLS-DA confirmed CP elevated desirable compounds while reducing microbial off-flavors (indole, dimethyl tetrasulfide). Correlation analysis revealed a negative association between 1-octen-3-ol and lipolytic enzymes (*r* = −0.89, *P* < 0.05), suggesting enzymatic flavor regulation. From a practical standpoint, GasA-CP is recommended for preserving refrigerated tilapia with balanced flavor quality.

## Highlights

• CP suppressed LOX and lipases activities via RONS, altering lipid oxidation pathways.
• CP enriched desirable aldehydes (64% increase) and reduced microbial off-flavors.
• Low-oxygen CP best balanced flavor enhancement and off-flavor inhibition in tilapia.
• A strong enzyme-flavor correlation (r = −0.89) revealed the mechanism for aroma development.

## Graphical abstract

**Figure:**
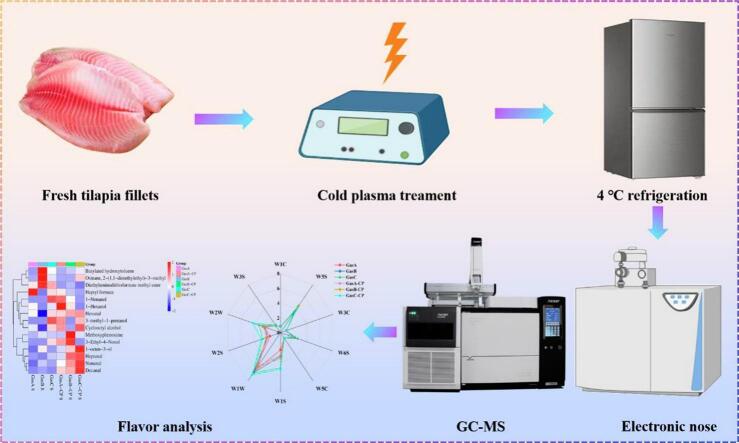

## 1 Introduction

The preservation of tilapia, a widely farmed freshwater fish, is challenging due to its high susceptibility to lipid oxidation and microbial spoilage, leading to rapid quality deterioration and undesirable fishy odors during refrigerated storage (Cai et al., 2024; Liu et al., 2023; Yang et al., 2025). Its fatty acid profile, rich in oleic (24–29%) and linoleic acids (20–24%), makes it highly susceptible to oxidation-derived flavor changes (Ullah et al., 2023). Traditional preservation methods, such as modified atmosphere packaging (MAP) and chemical antioxidants, have limitations in effectively controlling lipid oxidation while maintaining sensory quality (Miao et al., 2025; Zhao et al., 2025). Therefore, there is a growing need for innovative non-thermal technologies that can extend shelf life while preserving desirable flavor attributes.

Cold plasma (CP), an emerging non-thermal food processing technology, has gained attention for its ability to inactivate microorganisms and enzymes while modifying lipid oxidation pathways through reactive oxygen and nitrogen species (RONS) (Wang, Liu, et al., 2024). CP treatment influences flavor development by promoting volatile compounds from lipid peroxidation while suppressing microbial spoilage metabolites (Tappi et al., 2025; Wang, Zeng, et al., 2024). The composition of the excitation gas plays a crucial role in determining RONS production, which affects oxidative stability and flavor profile (Wu, Cui, & Li, 2023). For instance, high-oxygen CP enhances lipid oxidation and aldehyde formation, whereas nitrogen-rich CP may favor alternative pathways (Ke et al., 2021). However, how different CP excitation gas compositions modulate the dynamic evolution of key flavor compounds and the related enzyme activities in refrigerated tilapia remains unknown. While CP has been widely studied for its antimicrobial effects, its potential to influence flavor development through modulating lipid oxidation pathways has received less attention.

Previous studies have predominantly focused on the lipid oxidation of CP, with limited investigation into its impact on flavor chemistry, particularly in aquatic products (Sang et al., 2024). Key fish flavor compounds (hexanal, nonanal, 1-octen-3-ol) derive from enzymatic oxidation of PUFAs by lipoxygenase (LOX) and lipases. (Karbsri et al., 2024). CP-induced RONS may modulate these pathways via direct structural inactivation or altered substrate availability (Wang, Zhang, et al., 2022). Studies on Pacific white shrimp showed that Ar:Air plasma resulted in slower TBARS accumulation than Ar:O₂ plasma (Shiekh & Benjakul, 2020). Similar trends were observed in poultry and meat, where low-oxygen CP minimized lipid oxidation (Huang et al., 2018; Zhao et al., 2022). Additionally, CP effectively suppresses microbial off-flavors such as indole and sulfides (Qiu et al., 2024). These findings highlight that the excitation medium is a key determinant of CP-induced oxidative reactions, and low-oxygen conditions may balance flavor enhancement with oxidative stability.

Therefore, this study specifically investigated the effects of CP generated with different gas compositions (O_2_/N_2_/CO_2_ ratios) on LOX and lipase activities, as well as the formation and dynamic evolution of key volatile organic compounds (VOCs) in refrigerated tilapia fillets. By integrating electronic nose analysis, GC–MS, and multivariate statistical modeling, we evaluated the correlation between enzymatic inhibition and flavor development, identified dominant flavor contributors, and elucidated the CP-mediated flavor regulation mechanism. Unlike previous studies focusing on CP's antimicrobial effects or single-gas systems, this study provides three novel contributions: systematic comparison of gas compositions for flavor modulation, integration of GC–MS/OAV/multivariate analysis to link CP parameters to flavor compounds, and correlation analysis between volatiles and enzyme activities. These findings establish a scientific basis for optimizing CP as a flavor-oriented preservation technology.

## 2 Materials and methods

### 2.1 Materials and chemicals

Fresh tilapia fillets were sourced from Hainan Quanyi Food Co., Ltd. (Haikou, China). High-performance liquid chromatography (HPLC)-grade methanol and acetonitrile were obtained from Sigma-Aldrich (St. Louis, MO, USA). All other chemicals and reagents used were of analytical grade and employed without further purification.

### 2.2 Sample preparation

The dielectric barrier discharge (DBD) system (BK130/36, Phoenix Electric Co. Ltd., USA) was employed to perform CP treatment on tilapia fillets following our previous protocol (Sang et al., 2024). Briefly, tilapia fillets were randomly allocated into three experimental groups with distinct modified atmosphere packaging gas compositions: GasA (10% O₂, 50% N₂, 40% CO₂), GasB (air: 22% O₂, 78% N₂), and GasC (30% O₂, 30% N₂, 40% CO₂). The selection of these three gas compositions was based on the following considerations: GasA (low-oxygen, high-CO₂) represents a commonly used modified atmosphere packaging formulation for aquatic products, where elevated CO₂ levels effectively inhibit the growth of psychrotrophic spoilage bacteria; GasB (air) served as a control simulating conventional aerobic packaging conditions; GasC (high-oxygen, high-CO₂) was widely applied in the seafood industry to maintain bright red color of fish fillets while providing antimicrobial effects through CO₂. These formulations were chosen to cover a range of oxygen availability (from 10% to 30%) and CO₂ levels (0% to 40%), enabling systematic evaluation of how excitation gas composition modulates CP characteristics and subsequent effects on flavor quality. All three gas compositions are commercially feasible and have been validated in industrial modified atmosphere packaging operations for aquatic products. The DBD system was operated at a frequency of 120 kHz, with an electrode-to-sample distance of 5 mm. The plasma parameters (70 kV, 3 min) were selected based on preliminary optimization as they effectively generate reactive species without excessive lipid oxidation or thermal damage, consistent with our previous protocol (Wang, Fu, et al., 2022). All packaged samples underwent plasma treatment at 70 kV for 3 min before being stored at 4 °C. During 8 d storage period, samples from each group were analyzed at 2 d intervals. A non-plasma-treated control group with identical packaging was processed and sampled in parallel for comparative analysis.

### 2.3 Electronic nose measurement

The volatile components of the samples were analyzed using an electronic nose system (PEN3.0, Airsense Analytics Co. Ltd., Germany). Briefly, 5.0 g of homogenized fish sample was weighed into a 50 mL beaker and immediately sealed with three layers of plastic film. After equilibration at 25 °C for 15 min, the sample was analyzed using the electronic nose equipped with 10 metal oxide sensors. Seven sensors (W1S, W3S, W5S, W6S, W1C, W3C, and W5C) exhibited specific responses to various volatile compounds, including short-chain alkanes, long-chain alkanes, nitrogen oxides, hydrogen, and aromatic hydrocarbons (detailed sensor specifications are provided in Table 1). The operational parameters were set as follows: 80 s sensor cleaning time, 5 s auto-zeroing time, 5 s sample preparation time, 400 mL/min carrier gas flow rate, and 80 s sampling time. The stable response curves recorded between 70 and 72 s were selected for data analysis. This analytical procedure was adapted from the method described by Jin et al. (2023) with minor modifications.

**Table 1:** Performance description of electronic nose sensors.

No.	Sensor name	Performance description
1	W1C	Sensitive to aromatics
2	W5S	High sensitivity and sensitive to nitrogen oxides
3	W3C	Sensitive to ammonia and aromatic compounds
4	W6S	Mainly selective for hydrides
5	W5C	Short-chain alkane aromatic component
6	W1S	Sensitive to short-chain alkanes
7	W1W	Sensitive to organic sulfides and terpenes
8	W2S	Sensitive to alcoholes, aldehydes and ketones
9	W2W	Sensitive to aromatic compounds, sulfur and chlorine compounds
10	W3S	Sensitive to long-chain alkanes

### 2.4 Determination of volatile organic compounds (VOCs)

A 2 g aliquot of minced tilapia was homogenized with 2 mL of saturated NaCl solution and transferred to a 20 mL headspace vial. For quantification, 1 μL of the internal standard (2,4,6-trimethylpyridine, 2 μg/μL) was spiked into each vial, resulting in a final concentration of 1000 μg/kg in the sample. Volatile compounds were extracted using a 50/30 μm DVB/CAR/PDMS fiber (Supelco, Bellefonte, PA, USA), which was conditioned at 280 °C for 40 min in the GC–MS injection port before use. During analysis, the fiber was exposed to the vial headspace at room temperature for 40 min, followed by thermal desorption in the GC injector at 280 °C for 10 min.

Flavor compounds were analyzed by GC–MS using a 30 m × 0.25 mm × 0.25 μm capillary column (J&W Scientific, Folsom, CA, USA) (Qiu et al., 2024). Helium carrier gas was delivered at a constant flow rate of 0.8 mL/min in splitless mode, with the injector maintained at 250 °C. The oven temperature program was as follows: 40 °C (hold 4 min), ramped at 6 °C/min to 70 °C, then at 8 °C/min to 240 °C (hold 5 min). MS detection was performed in electron ionization (EI) mode at 70 eV, with the ion source and transfer line temperatures set to 230 °C and 250 °C, respectively. Mass spectra were acquired over the *m*/*z* range 50–350. Compound identification was performed by comparing mass spectra with the NIST library and retention indices with published data. Semi-quantitative analysis was conducted using the internal standard method without absolute quantification.

### 2.5 Calculation of odor activity values (OAVs)

The key aroma-active compounds were identified based on OAVs, which were calculated as the ratio between the concentration of each volatile compound and its corresponding odor threshold (Feng et al., 2024). The OAV was determined using the following equation:

OAV = C_i_ / T_i_.

where C_i_ represents the concentration of the compound in the sample (μg/kg), and T_i_ denotes the odor threshold of the compound (μg/kg). The odor threshold values were obtained from the published literature (van Gemert, 2011), and all thresholds are reported as water-based values, as commonly used for fish matrix analysis.

Compounds with OAV ≥ 0.1 were considered to have a modifying effect on the overall flavor profile, while those with OAV ≥ 1 were regarded as making a significant contribution to the overall flavor. Generally, the higher the OAV of a volatile compound, the greater its impact on the overall flavor characteristics.

### 2.6 Statistical analysis

All experiments were performed in triplicate, with results presented as mean ± standard deviation. Statistical analysis was conducted using SPSS 20 (SPSS Inc., Chicago, IL, USA), where one-way analysis of variance (ANOVA) followed by Duncan's multiple range test was applied to determine significant differences among groups (*P* < 0.05). Orthogonal partial least squares-discriminant analysis (OPLS-DA) and corresponding model permutation tests were performed using SIMCA 14.1 (Sartorius AG, Göttingen, Germany). The OPLS-DA models were validated using 200 permutation tests to confirm model validity and avoid overfitting. Data visualization was generated using Origin 2022 (OriginLab Corp., Northampton, MA, USA), while heatmap analysis was carried out with TBtools software (v2.034).

## 3 Results and discussion

### 3.1 Electronic nose analysis

**Fig. 1:**
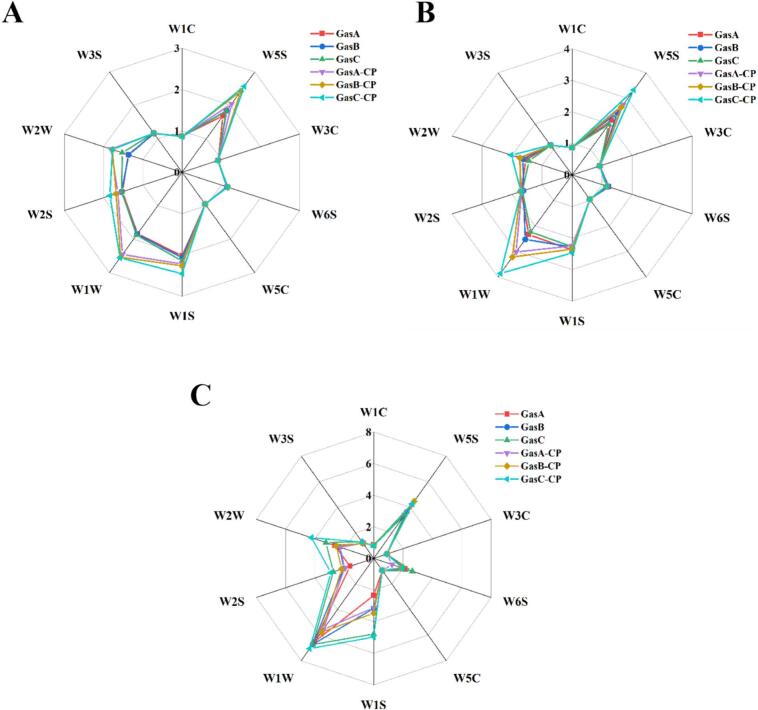
Radar plots of electronic nose responses for tilapia fillets subjected to different treatments after 0 d (A), 4 d (B), and 8 d (C) of refrigerated storage.

The relative resistivity (G/G0) values obtained from electronic nose sensors reflect the intensity of responses to volatile compounds, with higher values indicating stronger detection signals. However, these sensor responses represent a composite fingerprint of volatile profiles rather than direct quantification of specific flavor compounds. As shown in Fig. 1A-C, at day 0 of refrigeration, CP-treated tilapia fillets exhibited notably higher response values in sensors W1W (sensitive to inorganic sulfides), W5S (highly sensitive to nitrogen oxides), W1S (sensitive to short-chain alkanes), W2S (sensitive to alcohols, ethers, aldehydes and ketones), and W2W (sensitive to organic sulfides and aromatic compounds), while other sensors showed minimal and comparable responses. Particularly, the CP-treated samples demonstrated significantly elevated signals in W1W, W5S, and W2W sensors compared to control (*P* < 0.05), suggesting the generation of odor-active compounds including ozone, NO, and NO_2_ during CP treatment. These compounds specifically enhanced sulfur- and nitrogen oxide-sensitive sensors. Similar observations were reported in CP-treated goat milk (Wang, Liu, et al., 2022). Although CP treatment increased the electronic nose responses to sulfides and nitrogen oxides, these sensor signals primarily indicate changes in volatile fingerprints rather than directly representing perceived off-flavors. Therefore, GC–MS analysis was further employed to quantitatively identify the specific compounds responsible for flavor perception in tilapia fillets. Notably, the significant increase in W2S sensor values of treated samples indicated that CP processing promoted the formation of alcohols, ethers, aldehydes and ketones in fish flesh (*P* < 0.05). During prolonged refrigeration, untreated samples exhibited rising W1W values, suggesting microbial and enzymatic production of off-flavor indoles and sulfides from protein and lipid oxidation. By 8 d of storage, there was no significant difference in W1W sensor responses between treated and untreated groups (*P* > 0.05), indicating the gradual diminishment of CP's flavor-preserving effects. Overall, the electronic nose data revealed treatment-dependent differences in volatile fingerprints, which were further resolved by GC–MS to identify specific flavor-active compounds (Section 3.2).

### 3.2 VOCs analysis

VOCs were identified by GC–MS as described in Section 2.4. Compound identification was based on matching mass spectra with the NIST library and comparison of retention indices with published data. Due to the large number of samples and the qualitative nature of this analysis (66 compounds across multiple treatments and time points), presenting full chromatograms and mass spectra for all compounds in the main manuscript would be impractical and uninformative. Instead, the complete list of identified compounds with their relative concentrations is provided in Supplementary Table S1, and key patterns are visualized through heatmaps (Fig. 3A), OPLS-DA score plots (Fig. 4), and variable importance in projection (VIP) analysis (Fig. 3B), which more effectively convey the treatment-dependent differences in volatile profiles.

#### 3.2.1 Analysis of VOCs composition

**Fig. 2:**
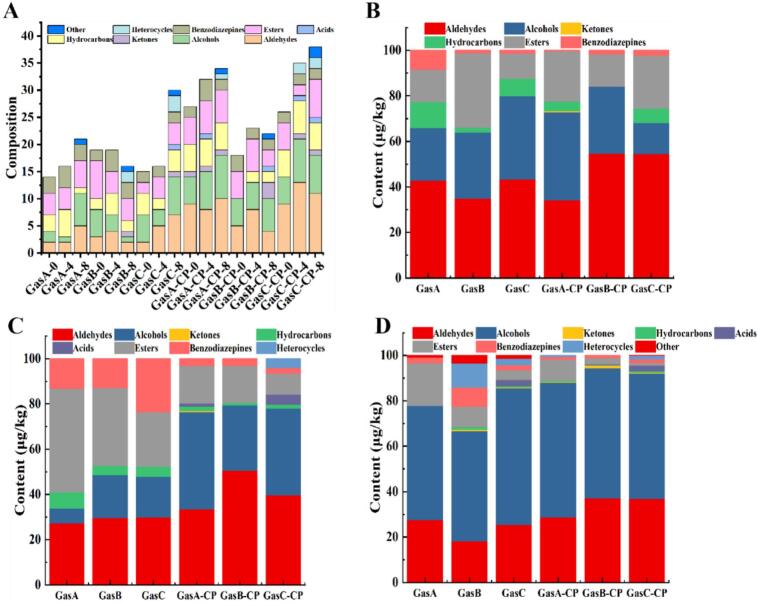
The types of volatile organic compounds (A) in tilapia fillets after different treatments during refrigeration and their relative content changes at 0 d (B), 4 d (C), and 8 d (D).

A total of 66 volatile flavor compounds were identified in tilapia during refrigerated storage (Table S1), which were categorized into 9 classes: 15 aldehydes, 15 alcohols, 4 ketones, 7 hydrocarbons, 2 acids, 13 esters, 4 benzodiazepines, 3 heterocycles, and 3 others. As shown in Fig. 2A, the variety of volatile compounds increased progressively with refrigeration time. CP-treated samples exhibited greater diversity and higher concentrations of volatile compounds, particularly aldehydes and alcohols, which were most closely associated with flavor changes in tilapia fillets. Although hydrocarbons, esters, and benzodiazepines showed abundant varieties, their profiles remained relatively stable during storage. Notably, several ketones, acids, and other compounds were only detected in later storage stages and treated groups, indicating that CP treatment effectively enriched the volatile flavor profile of tilapia (Qiu et al., 2024).

The relative abundance distribution of volatile compounds revealed significant differences among the 9 classes during refrigeration (*P* < 0.05) (Fig. 2B-D). Aldehydes, alcohols, and esters constituted the dominant flavor components, showing significantly higher levels than other compounds (*P* < 0.05). At 0 d, alcohols and aldehydes collectively accounted for over 60% of total volatile compounds. As storage progressed to 4 d, ester levels exhibited a decreasing trend across all treatment groups, with GasC-CP showing a more pronounced reduction compared to GasA-CP and GasB-CP. Simultaneously, aldehyde and alcohol contents increased across all treated samples. At 8 d, all groups displayed significantly elevated alcohol levels compared to initial stages (*P* < 0.05), with decreased yet still predominant aldehyde contents. Aldehydes and alcohols maintained approximately 10% relative abundance in early storage, while other volatiles, despite their low concentrations, still contributed to the overall flavor profile of tilapia fillets.

**Fig. 3:**
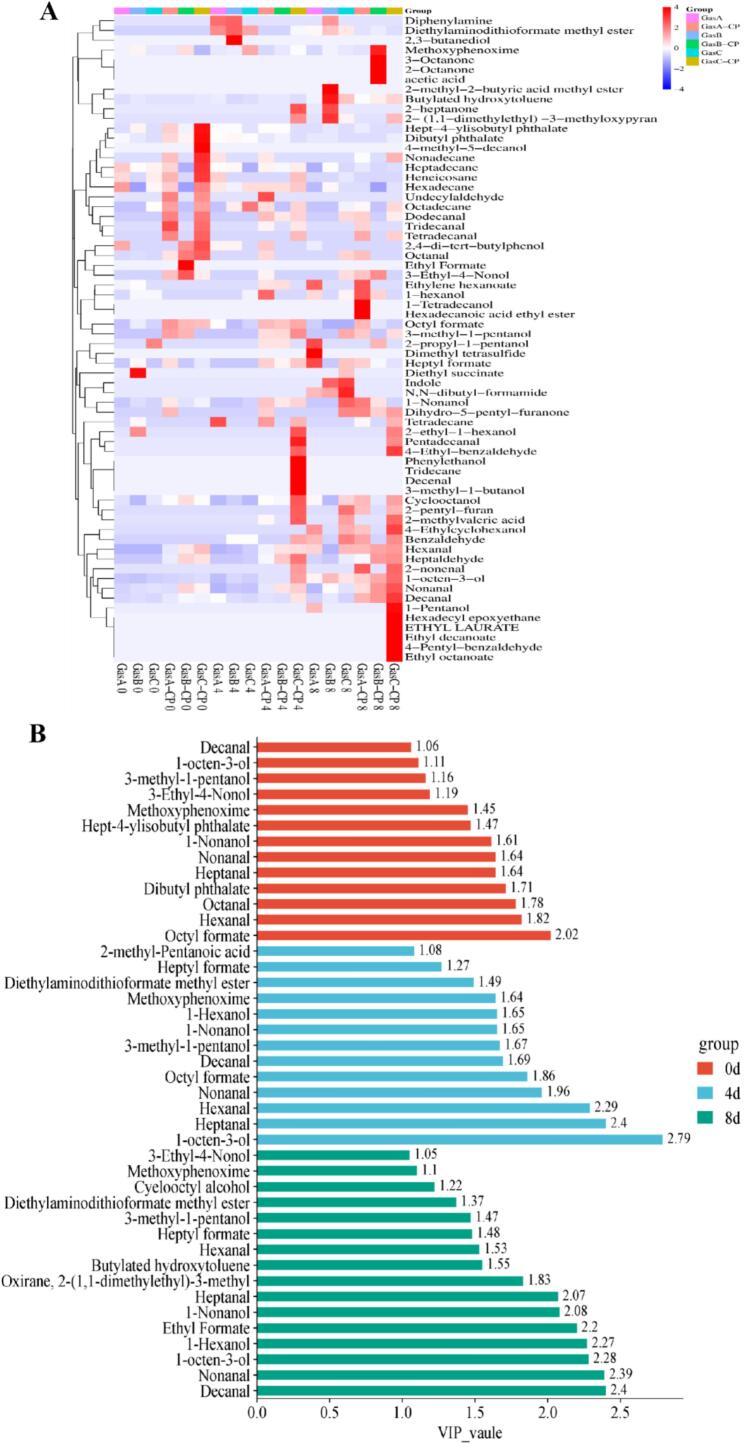
The heat maps of volatile organic compounds detected in all refrigerated tilapia samples identified by GC/MS (A). Volatile organic compounds with VIP score > 1 identified through GC/MS (B).

Thermographic clustering analysis was employed to investigate volatile flavor compound differences in tilapia chunks across treatment groups during storage (Fig. 3A). Aldehydes, identified as primary flavor contributors, exhibited characteristic low threshold values and distinct fatty aromas, originating predominantly from unsaturated fatty acid oxidation and amino acid degradation (Wu et al., 2022). The detected aldehydes were hexanal, heptanal, octanal, and nonanal, mainly derived from the oxidative degradation of linolenic acid, linoleic acid, and oleic acid (Al-Dalali et al., 2021). In our previous study (Sang et al., 2024), we reported the fatty acid composition of Nile tilapia under different temperature and packaging conditions, specifically the contents of oleic acid (C18:1), linoleic acid (C18:2n-6), and linolenic acid (C18:3n-3). These fatty acid ranges (oleic 24.45–28.75%, linoleic 20.40–24.22%) are consistent with those reported by Ullah et al. (2023). Such fatty acids served as the primary precursors for the formation of hexanal, nonanal, heptanal, and 1-octen-3-ol via enzymatic (LOX/lipase) and non-enzymatic lipid oxidation pathways. Briefly, the high oleic acid content in tilapia yields various aldehydes via cleavage of oxidation products, while β-scission of linoleic acid-derived alkoxy radicals generates hexanal (Al-Dalali et al., 2024; Wang et al., 2025). Consistent with this, the GasC-CP group showed the greatest decline in polyunsaturated fatty acids and the highest aldehyde diversity, whereas the GasA-CP group better preserved n-3 and n-6 fatty acids and exhibited a more balanced volatile profile (Sang et al., 2024). Untreated samples contained only octanal, nonanal, and decanal, whereas CP-treated samples showed 9 aldehydes with significantly elevated total content (*P* < 0.05), consistent with findings in dry-cured black carp (Ke et al., 2021). The GasC-CP group exhibited 6 additional aldehydes after 4 d refrigeration, including benzaldehyde (imparting nutty/fruity notes) and characteristic 2-octenal/2-nonenal from linoleic/linolenic acid oxidation (Karbsri et al., 2024). By 8 d, all treatments demonstrated progressive aldehyde accumulation, particularly for hexanal, benzaldehyde, nonanal, and decanal (*P* < 0.05). The GasC-CP group maintained the highest aldehyde diversity and concentration, attributable to ROS/RNS-mediated lipid peroxidation that generated unstable hydroperoxides and subsequent aldehyde formation (Chen, Fan, et al., 2023). This effect was enhanced under high-oxygen conditions, ultimately improving overall flavor profiles through increased aldehyde variety and concentration (Shiekh et al., 2021).

Alcohols constitute a major category of volatile compounds in fish products. Compared to their saturated counterparts, unsaturated alcohols demonstrate lower odor thresholds and contribute more substantially to flavor development through their characteristic mild and oily notes (Sun et al., 2022). 15 volatile alcohols were identified during refrigeration storage, with 1-octen-3-ol, 1-hexanol, 3-methyl-1-pentanol, cyclooctanol, and 1-nonanol were the main species. As a key flavor component of fresh fish, 1-octen-3-ol exhibited distinctive mushroom and earthy aroma, mainly produced through the metabolic breakdown of arachidonic acid and linoleic acid (Zhang et al., 2022). Notably, 1-hexanol, a known contributor to undesirable fishy odors, was exclusively detected in control samples at 0 d, suggesting that CP treatment effectively suppressed its formation. The subsequent emergence of 1-hexanol during storage might be due to progressive lipid degradation. Other detected alcohols, mainly derived from unsaturated fatty acid oxidation, played supplementary roles in flavor modulation (Yin et al., 2020). CP-treated samples initially contained slightly higher 1-octen-3-ol concentrations, which increased significantly after 4 d of refrigeration (*P* < 0.05). In contrast, control samples showed comparable increases only after 8 d. This temporal pattern corroborates findings by Chu et al. (2023) in refrigerated grouper, where advancing lipid oxidation accelerated 1-octen-3-ol formation. Among CP treatments, the GasC-CP group (high-O₂ atmosphere) displayed significantly greater 1-octen-3-ol accumulation than GasB-CP and GasA-CP groups (*P* < 0.05), confirming the enhanced lipid oxidation under oxygen-rich conditions. During extended storage, total alcohol concentrations increased substantially (*P* < 0.05), with CP-treated samples maintaining marginally higher levels than controls. Given the established role of alcohols in shaping characteristic fatty flavors (Chu et al., 2023), the CP-induced elevation of these compounds may contribute to the development of unique flavor profiles in fish.

Ketones, which exhibit relatively high odor thresholds compared to other volatile compounds, are primarily formed through oxidative degradation of unsaturated fatty acids and alcohols, or via secondary reactions including alcohol oxidation and ester decomposition (Wang et al., 2021). Although their direct contribution to overall fish flavor was limited due to these higher thresholds, ketones have been shown to synergistically enhance flavor perception. After 8 d of refrigeration, 4 ketones were mainly identified in tilapia fillets, namely 2-heptanone, 3-octanone, 2-octanone, and dihydro-5-pentyl-furanone. 2-heptanone, a known oxidation product of linoleic acid, contributed characteristic meaty and gravy-like aromatic notes (Tan et al., 2022). Both 3-octanone and 2-octanone, generated through autoxidation of linoleic and linolenic acids, were found to impart distinct fruity aromas (Zhang et al., 2023). While dihydro-5-pentyl-furanone was consistently detected in treated samples during later storage periods, its specific sensory properties remain poorly characterized in the scientific literature. Therefore, the observed increase in ketone concentrations in treated samples during extended refrigeration might help counterbalance the fishy notes associated with accumulated aldehydes and alcohols.

Alkanes constitute an important class of volatile organic compounds prevalent in aquatic food products, particularly crustaceans and fish (Li et al., 2023). These compounds function as crucial intermediates in heterocycle formation and contribute to flavor development through their distinctive alkaline characteristics. A progressive decline in hydrocarbon content throughout refrigerated storage in both control and treated tilapia fillets (*P* < 0.05). The formation of these alkanes principally occurs via homolytic cleavage of fatty acid alkoxy radicals (Zhang et al., 2024). Through lipid oxidation pathways, long-chain alkanes undergo fragmentation into smaller molecular species, while alkenes may subsequently transform into aldehydes and ketones given suitable conditions (Xue et al., 2024). Despite their characteristically high odor thresholds, the detected long-chain hydrocarbons in refrigerated tilapia were found to impart desirable sweet aromas that positively influenced overall flavor perception (Qiu et al., 2024). Notably, after 8 days of refrigeration, CP-treated samples maintained higher alkane concentrations compared to untreated group. This suggested that cold plasma treatment might facilitate the retention or formation of favorable flavor-active hydrocarbons. The preservation of these hydrocarbon compounds could be attributed to CP-induced modifications in lipid oxidation pathways, potentially through stabilization of alkoxy radicals or alteration of secondary reaction products.

Acids were detected at minimal levels in refrigerated tilapia fillets, primarily associated with hydrolytic rancidity or fatty acid oxidation. During the refrigeration process, only two short chain acids were identified in the processed samples, namely acetic acid and 2-methylvaleric acid. These volatile short-chain acids are known to contribute pungent, rancid odors while simultaneously serving as precursors for ester formation, particularly acetic acid (Chen, Zhang, et al., 2023). Notably, due to its low odor threshold, acetic acid played a significant role in flavor development during refrigerated storage.

Ester formation in fish systems occurs through two principal biochemical pathways: esterification between free fatty acids and alcohols, and alcoholysis via triglyceride-ethanol transesterification reactions. Lactones, which constitute a distinct class of cyclic esters generated through *β*-oxidation of unsaturated fatty acids, are particularly notable for their characteristic fruity and creamy aromatic profiles. These compounds demonstrate dual functionality by both neutralizing undesirable odors derived from fatty acid degradation products and amino compound-related bitter flavors, while simultaneously imparting favorable sensory attributes at low concentrations (Wang, Zhang, et al., 2022). 4 dominant ester species during refrigerated storage: heptyl formate, octyl formate, diethylaminodithioformate methyl ester, and dibutyl phthalate. Immediate effects of CP treatment included significant increases in octyl formate, hept-4-yl isobutyl phthalate, and dibutyl phthalate concentrations at 0 d (*P* < 0.05), attributable to CP-induced lipid oxidation that elevated precursor availability for esterification. Of particular interest was the exclusive detection of ethyl formate in treated samples, which might be the result of formic acid oxidation conversion (Zou et al., 2025). Throughout 8 d storage period, a general decline in ester levels was observed across all groups. However, the residual ester content in the GasA-CP treatment group was higher than that in other treatment groups and the control (*P* < 0.05). This persistent ester retention suggests a potential mechanism for off-flavor suppression and quality preservation in refrigerated fish products.

Benzodiazepines derivative detected in refrigerated tilapia fillets primarily included methoxyphenoxime and butylated hydroxytoluene (BHT). Methoxybenzoxime concentrations exhibited a progressive decline during storage. Although this environmental contaminant has been detected in various meat products, our sensory evaluation confirmed its negligible contribution to fish flavor profiles. BHT demonstrated a significant reduction by 8 d, consistent with the complete disappearance reported by Cheng et al. (2023) during late storage phases. As an odorless, tasteless compound, BHT minimally impacts flavor development. Heterocycles predominantly originate from Maillard reactions between reducing sugars and amino acids, amino acid pyrolysis, and thiamine degradation (Zhu et al., 2023). The emergence of amine compounds, particularly diphenylamine detected in controls at 4 d and N, N-dibutyl-formamide (peaking at 0.8 μg/kg in GasC group), signaled declining freshness through characteristic fishy odors. Notably, 2-pentyl-furan concentrations (derived from linoleic acid oxidation) were higher in CP-treated groups (*P* < 0.05), with GasA-CP showing maximal retention. While this compound imparts fruity/cheesy notes, olfactometry results confirmed furans contribute minimally to overall aroma profiles (Zhang et al., 2022; K. Zhang et al., 2021). The trace presence of 2-(1,1-dimethylethyl)-3-methyloxypyran showed no significant sensory impact (*P* > 0.05). Microbial spoilage markers became evident by 8 d, with control groups (GasB/GasC) accumulating indole and dimethyl tetrasulfide, which were volatile compounds associated with protein degradation (Ma et al., 2021). Their absence in CP-treated samples demonstrated the CP's antimicrobial efficacy, preserving desirable flavor characteristics throughout refrigerated storage.

This study comprehensively analyzed the volatile flavor profiles of refrigerated tilapia fillets under different CP treatment conditions. CP processing significantly enhanced the formation of key flavor compounds, particularly aldehydes (hexanal, nonanal) and unsaturated alcohols (1-octen-3-ol), through promoted lipid oxidation and modified degradation pathways of unsaturated fatty acids (oleic, linoleic, and linolenic acids). High-oxygen CP treatments (GasC-CP) exhibited the most pronounced effects, yielding greater diversity and concentration of volatile compounds, which contributed to improved flavor quality. Ketones and esters, though less impactful individually, synergistically enhanced flavor profiles by masking undesirable odors and providing fruity/creamy notes. CP treatment also effectively suppressed the formation of off-flavor compounds (amines, indole, and sulfides) associated with microbial spoilage and protein degradation, thereby extending sensory shelf life. In contrast, untreated samples accumulated higher levels of fishy/rancid volatiles during storage. These findings demonstrate that CP technology can optimize flavor development in refrigerated fish by modulating lipid oxidation pathways while simultaneously inhibiting spoilage-related odorants. The results provide valuable insights for applying CP as a novel preservation strategy to maintain or enhance the sensory quality of aquatic products.

#### 3.2.2 Key VOCs

**Fig. 4:**
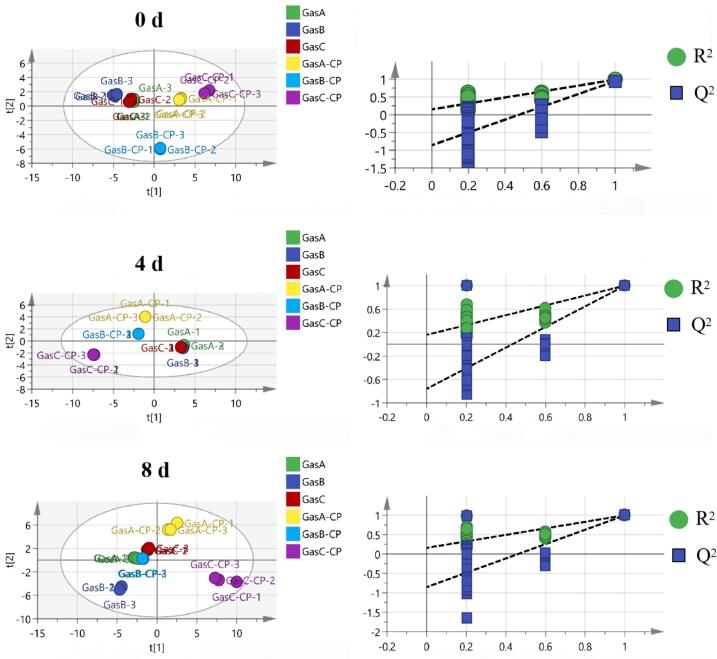
Multivariate analysis of volatile profiles by OPLS-DA and model validation using GC/MS data across refrigeration conditions.

OPLS-DA effectively discriminated volatile compound profiles among treatment groups (Wang et al., 2023). VOCs data from 0, 4, and 8 d of refrigeration were Pareto-scaled and analyzed using OPLS-DA (Fig. 4). The models demonstrated excellent goodness-of-fit and predictive capability, with parameters for 0 d (R^2^X = 0.962, R^2^Y = 0.890, Q^2^ = 0.803), 4 d (R^2^X = 0.988, R^2^Y = 0.970, Q^2^ = 0.881), and 8 d (R^2^X = 0.974, R^2^Y = 0.922, Q^2^ = 0.504). The R^2^Y values (89.0–97.0%) and Q^2^ > 0.5 collectively indicated high model reliability (Wu, Wang, et al., 2023). Model validity was confirmed by 200 permutation tests (as described in Section 2.6).

Score plot visualization revealed distinct sample clustering patterns (Fig. 4). CP-treated samples primarily localized in quadrants II and IV, while control groups clustered in quadrant I, demonstrating significant (*P* < 0.05) flavor profile differentiation. At 0 d, control groups (GasA/B/C) showed overlapping distributions, indicating minimal initial variation, whereas CP-treated groups exhibited clear spatial separation along the predictive component axis, suggesting gas composition-dependent flavor modulation. By 4 d, GasC-CP (high-O₂) samples showed maximal separation from other groups. At refrigeration endpoint (8 d), all treatment groups became well-distinguished, with GasA-CP (low-O₂) maintaining stable positioning near quadrant boundaries, demonstrating superior flavor preservation capacity.

**Fig. 5:**
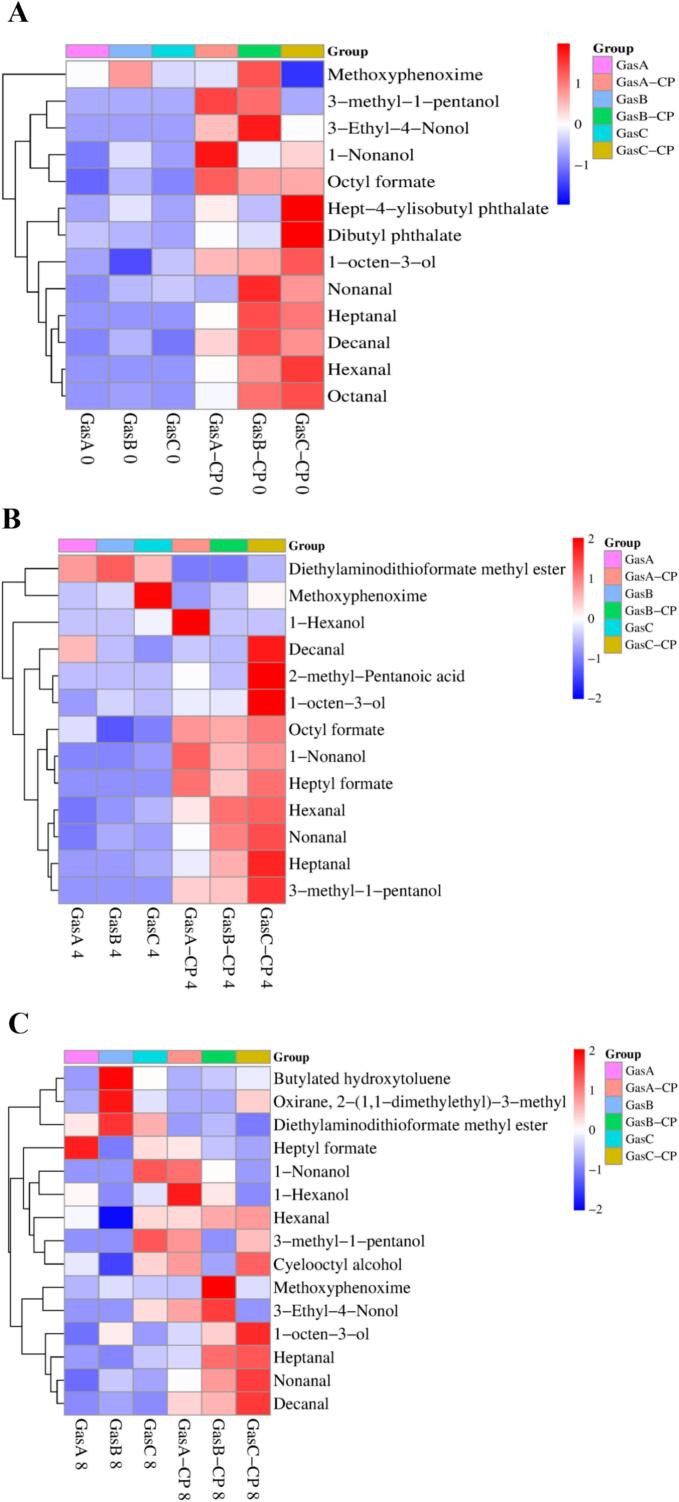
Cluster heatmap of differential markers during refrigeration of tilapia fillets. High concentration is shown in red, low concentration in blue (see color scale). (For interpretation of the references to color in this figure legend, the reader is referred to the web version of this article.)

VIP analysis (threshold >1.0) identified 20 discriminant flavor compounds (Fig. 3B), comprising: 5 aldehydes (including hexanal and nonanal), 6 alcohols (notably 1-octen-3-ol), 1 acid, 5 esters, and 3 aromatic compounds. The 20 discriminant compounds with their VIP values are listed in Table 3. Heatmap visualization confirmed significantly elevated concentrations of lipid oxidation-derived aldehydes and alcohols in CP-treated samples (*P* < 0.05) (Fig. 5), consistent with RONS-mediated peroxidation pathways. The temporal convergence of GasA-CP and control group flavor profiles by 8 d suggested attenuated oxidative effects under low-O₂ conditions. Notably, the exclusive detection of diethylaminodithioformate methyl ester in control groups implied CP-mediated suppression of microbial metabolic pathways.

### 3.3 Changes in OAVs values of key VOCs

Odor perception intensity is fundamentally determined by the combined effect of volatile compound concentrations and their individual sensory thresholds (Feng et al., 2024). The OAV, calculated as the ratio of compound concentration to its detection threshold, provides a quantitative measure of aroma contribution (Table 2). 8 flavor-active compounds with OAVs greater than 1.0 were identified in tilapia fillets: hexanal, heptanal, octanal, nonanal, decanal, 1-hexanol, 1-octen-3-ol, and 1-nonanol. These compounds collectively characterize tilapia's flavor profile, contributing sweet, citrusy, fatty, and fruity peel notes based on sensory correlation analysis (Li et al., 2025). Among these, hexanal, heptanal, nonanal, and 1-octen-3-ol are considered desirable flavor contributors at moderate concentrations; however, excessive accumulation of hexanal and heptanal leads to undesirable fishy odors (Fu et al., 2023). CP treatment significantly elevated aldehyde concentrations while maintaining favorable OAV ranges. The GasA-CP group optimally regulated these compounds, achieving desirable levels of hexanal (OAV = 1.59 at day 8) and heptanal (OAV = 0.54, below the off-odor threshold), significantly lower than other treatments (*P* < 0.05). This reflected low-O₂ conditions' dual capacity to both enhance desirable flavors and suppress hexanal-mediated off-odors (Huang et al., 2023).

**Table 2:** Odor activity values of dominant volatile organic compounds in refrigerated tilapia fillets.

No.	Compounds	Storage time (d)	Odor thresholds (μg/kg)	OAV					
				GasA	GasB	GasC	GasA-CP	GasB-CP	GasC-CP
1	Hexanal	0	4.5	–	–	–	0.57	1.16	1.61
		4	–	–	0.14	0.27	0.7	1.21	1.28
		8	–	1.38	–	1.66	1.59	1.94	2.02
2	Heptaldehyde	0	3	–	–	–	0.44	1.16	0.98
		4	–	–	–	0.1	0.56	1.26	2.23
		8	–	0.14	–	0.46	0.54	1.78	1.91
3	Octanal Octanal	0	0.7	–	–	–	1.97	5.31	5.59
		4	–	–	–	–	2.17	–	–
		8	–	–	–	2.37	2.84	–	–
4	Nonanal	0	1	2.46	2.92	3.03	2.81	5.49	4.49
		4	–	1.39	2.06	1.92	3.25	5.08	5.83
		8	–	1.57	3.88	3.01	5.27	7.79	10.13
5	Decana	0	1	0.22	0.36	0.17	0.74	1.17	0.95
		4	–	1.32	0.44	0.15	0.51	0.4	2.33
		8	–	0.07	0.67	0.06	3.91	4.76	7.63
6	1-hexanol	0	40	–	0.01	0.01	–	–	–
		4	–	–	–	0.01	0.09	–	–
		8	–	0.04	0	0.03	0.1	0.04	–
7	1-octen-3-ol	0	1	1.24	0.93	1.35	1.81	1.87	2.16
		4	–	0.64	1.92	1.47	2.59	2.43	8.69
		8	–	4.49	7.55	5.26	6.49	8.2	11.46
8	1-Nonanol	0	2	–	0.15	0.05	0.57	0.18	0.27
		4				0.06	0.82	0.57	0.69
		8				3.04	2.71	1.18	0.08

“-” Means VIP<1.

**Table 3:** Discriminant volatile compounds identified by VIP analysis (VIP > 1.0).

No.	Compound	VIP value
1	Hexanal	2.32
2	Nonanal	2.14
3	1-octen-3-ol	2.04
4	Heptanal	1.91
5	Octanal	1.85
6	1-Nonanol	1.75
7	Decanal	1.66
8	1-Hexanol	1.50
9	Octyl formate	1.48
10	Heptyl formate	1.37
11	Dibutyl phthalate	1.31
12	2-Heptanone	1.26
13	2-Octanone	1.24
14	Methoxyphenoxime	1.20
15	Butylated hydroxytoluene	1.19
16	Nonadecane	1.15
17	Hexadecane	1.11
18	Heptadecane	1.07
19	2-Methylvaleric acid	1.05
20	2-Pentyl-furan	1.01

Alcohol profiling revealed three key compounds: 1-hexanol, 1-octen-3-ol, and 1-nonanol. 1-octen-3-ol is a highly desirable flavor compound, imparting mushroom and earthy notes, while 1-hexanol contributes marginally to fishy off-notes when present at higher levels. 1-octen-3-ol's consistently high OAVs and intense mushroom/vegetal aromas establish it as a flavor-dominant compound. 1-Nonanol's fruity/fatty characteristics were particularly pronounced in GasC groups, correlating with lipid oxidation markers (Li et al., 2023). Notably, the control samples exclusively contained undesirable microbial spoilage markers including dimethyl tetrasulfide and indole, which have exceptionally low odor thresholds and generate putrid odors (Ma et al., 2021). Pathogenic bacteria such as *Staphylococcus aureus* could produce off-flavors and other spoilage metabolites under stress conditions (Chan et al., 2021). Similarly, amine compounds like diphenylamine and N, N-dibutyl-formamide, indicative of protein degradation and freshness loss, appeared only in 4 d controls. These findings demonstrate CP's dual functionality: inhibiting microbial growth to minimize off-flavors while attenuating negative sensory effects of compounds, effectively extending flavor stability (Cai et al., 2024; Qiu et al., 2024).

### 3.4 Correlation analysis

**Fig. 6:**
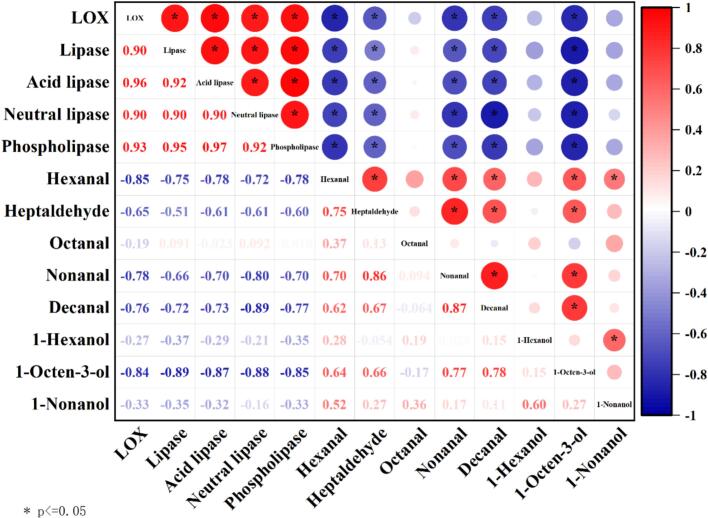
Correlation analysis of lipid oxidation value and key volatile flavor substances.

Volatile flavor compounds in refrigerated tilapia fillets were predominantly derived from lipid oxidative degradation pathways. To further investigate the enzymatic regulation of flavor development, we examined the impact of CP on lipid oxidation and lipase activity, building upon our previous study which demonstrated CP's efficacy in modulating these pathways. Pearson correlation analysis was subsequently performed between LOX activity, lipase profiles (including acid lipase, neutral lipase, and phospholipase), and key volatile compounds (Fig. 6). Hierarchical clustering of the resulting heatmap data effectively discriminated temporal flavor profiles across storage days (0, 4, and 8), highlighting distinct phase-dependent changes in volatile composition. Statistical analysis revealed significant positive correlations (*P* < 0.05) among all measured enzyme activities, confirming their cooperative action in lipid peroxidation and polyunsaturated fatty acid breakdown. Conversely, LOX activity demonstrated significant negative correlations with hexanal, heptanal, nonanal, and 1-octen-3-ol. Notably, 1-octen-3-ol showed the strongest inverse relationship with phospholipase activity (*r* = −0.89, *P* < 0.05). This negative correlation might arise from enzyme-substrate competition (redirecting lipid substrates) or RONS-mediated enzyme inactivation, reducing conversion of lipid hydroperoxides to 1-octen-3-ol. However, as direct measurements of CP-induced enzyme inhibition (activity assays) were not performed in this study, this interpretation remains correlational rather than causal. No significant associations were observed between enzymatic activities and octanal, 1-hexanol, or 1-nonanol concentrations, likely reflecting their subthreshold production levels (*P* > 0.05). Notably, the aldehyde compounds exhibited strong positive intercorrelations (hexanal-heptanal; heptanal-nonanal; nonanal-decanal), suggesting shared formation pathways via lipid oxidation cascades. The abundance of linoleic and oleic acids in tilapia explained the strong positive correlations among hexanal, heptanal, and nonanal, as these were specific oxidation products of these fatty acids (Ullah et al., 2023). These coordinated changes in volatile profiles ultimately shape the characteristic sweet, fatty, and citrus-like aroma notes in tilapia through combined oxidative and microbial metabolic processes.

## 4 Conclusion

GasA-CP provides the best balance between enhancing desirable aldehydes and suppressing fishy off-flavors. For tilapia preservation, a CP treatment with 10% O_2_/50% N_2_/40% CO_2_ is recommended to optimize flavor quality. This study provides novel mechanistic insights into CP-mediated flavor modulation in refrigerated tilapia, with three key contributions that have not been previously reported. First, we demonstrate for the first time that the composition of CP excitation gas critically determines the balance between desirable aldehyde/alcohol formation and off-flavor suppression, with GasA-CP (low-O₂) identified as optimal for flavor quality. Second, we established quantitative correlations (*r* > 0.9) among lipid-derived volatiles and negative associations with enzymatic activities (*r* < −0.8), providing new evidence for CP's targeted oxidative regulation of flavor pathways. Third, we performed OAV-based identification of 8 key flavor-active compounds, revealing that CP treatment selectively promotes desirable volatiles while eliminating microbial spoilage markers (indole, sulfides). While these findings demonstrate the potential of CP as a preservation technology, several limitations should be acknowledged, including the need for industrial scalability validation, potential economic challenges of CP equipment, and the necessity of sensory evaluation to assess consumer acceptability of CP-induced volatiles. Future studies should address these limitations through pilot-scale validation, cost-benefit analysis, and sensory evaluation. Further studies are needed to evaluate sensory acceptance by consumer panels and scale-up feasibility. Nonetheless, this work provides a scientific basis for further development of CP-based strategies to improve the sensory quality and stability of aquatic products during refrigerated storage.

## CRediT authorship contribution statement

**Tingting Yang:** Writing – original draft, Data curation. **Xiaohan Sang:** Methodology, Formal analysis. **Yuanyuan Wang:** Investigation. **Xueying Zhang:** Data curation. **Guanghua Xia:** Software. **Liming Zhang:** Writing – review & editing, Funding acquisition. **Jiamei Wang:** Project administration, Funding acquisition. **Yaqin Wang:** Resources. **Per Ertbjerg:** Writing – review & editing.

## Declaration of competing interest

The authors declare that they have no known competing financial interests or personal relationships that could have appeared to influence the work reported in this paper.

## Data Availability

Data will be made available on request.
