# Supplementary material for: Flavor evolution in refrigerated tilapia processed by cold plasma: Volatile profiles and odor quality driven by excitation gas-generated reactive species
Source: Food Chem X. 2026 Jun 17;37:104118. doi: 10.1016/j.fochx.2026.104118 (PMC13320458; doi:10.1016/j.fochx.2026.104118)
Supplement: Supplementary file 1 — Supplementary material [file mmc1.docx]

**Table S1** Changes in volatile organic compound content of tilapia fillets during refrigeration.

| No. | VOCs (μg/kg) | Rt/min | 0 d | | | | | | 4 d | | | | | | 8 d | | | | | |
| --- | --- | --- | --- | --- | --- | --- | --- | --- | --- | --- | --- | --- | --- | --- | --- | --- | --- | --- | --- | --- |
|  |  |  | GasA | GasB | GasC | GasA-CP | GasB-CP | GasC-CP | GasA | GasB | GasC | GasA-CP | GasB-CP | GasC-CP | GasA | GasB | GasC | GasA-CP | GasB-CP | GasC-CP |
| 1 | Hexanal | 5.984 | - | - | - | 2.55±0.05^c^ | 5.21±0.06^b^ | 7.24±0.09^a^ | - | 0.63±0.05^d^ | 1.21±0.03^c^ | 3.16±0.60^b^ | 5.45±0.12^a^ | 5.75±0.23^a^ | 6.22±0.20^d^ | - | 7.45±0.68^c^ | 7.45±0.46^c^ | 8.72±0.87^b^ | 9.10±0.78^a^ |
| 2 | Heptaldehyde | 10.948 | - | - | - | 1.32±0.06^c^ | 3.48±0.06^a^ | 2.94±0.03^b^ | - | - | 0.31±0.04^d^ | 1.69±0.12^c^ | 3.78±0.02^b^ | 6.69±0.77^a^ | 0.41±0.08^e^ | - | 1.38±0.10^d^ | 1.62±0.26^c^ | 5.33±0.41^b^ | 5.74±0.21^a^ |
| 3 | Octanal | 14.71 | - | - | 0.16±0.01^d^ | 1.38±0.16^c^ | 3.37±0.08^b^ | 3.91±0.22^a^ | - | - |  | 1.52±0.08^a^ | - | - | - | - | 1.66±0.23^b^ | 1.99±0.51^a^ | - | - |
| 4 | Nonanal | 17.359 | 2.46±0.11^e^ | 2.92±0.06^d^ | 3.03±0.03^c^ | 2.81±0.05^d^ | 5.49±0.07^a^ | 4.49±0.04^b^ | 1.39±0.11^f^ | 2.06±0.12^d^ | 1.92±0.15^e^ | 3.25±0.04^c^ | 5.08±0.12^b^ | 5.83±0.10^a^ | 1.57±0.30^f^ | 3.88±0.27^d^ | 3.01±0.11^e^ | 5.27±0.32^c^ | 7.79±0.20^b^ | 10.13±0.18^a^ |
| 5 | Decanal | 19.521 | 0.22±0.01^e^ | 0.36±0.01^d^ | 0.17±0.01^e^ | 0.74±0.06^c^ | 1.17±0.02^a^ | 0.95±0.26^b^ | 1.32±0.11^b^ | 0.44±0.01^d^ | 0.15±0.00^e^ | 0.51±0.01^c^ | 0.40±0.09^d^ | 2.33±0.10^a^ | 0.07±0.01^e^ | 0.67±0.09^d^ | 0.06±0.01e ^a^ | 3.91±0.c ^a^ | 4.76±0.23^b^ | 7.77±2.31^a^ |
| 6 | Undecylaldehyde | 21.419 | - | - | - | 0.18±0.03^a^ | - | 0.16±0.01^a^ | - | - | - | 0.24±0.02^a^ | - | - | - | - | - | - | - | - |
| 7 | Dodecanal | 23.082 | - | - | - | 0.26±0.03^a^ | - | 0.28±0.05^a^ | - | - | - | 0.17±0.02^a^ | 0.11±0.00^b^ | 0.14±0.01^a^ | - | - | 0.13±0.00^a^ | 0.15±0.02^a^ | - | 0.10±0.00^a^ |
| 8 | Tridecanal | 24.541 | - | - | - | 0.48±0.09^a^ | - | 0.40±0.03^b^ | - | - | - | 0.19±0.00^a^ | - | 0.12±0.01^b^ | - | - | - | 0.17±0.00^a^ | - | - |
| 9 | Tetradecanal | 25.855 | - | - | - | 0.18±0.01^a^ | - | 0.22±0.06^a^ | - | - | - | - | - | 0.12±0.01^a^ | - | - | - | 0.14±0.04^a^ | - | 0.10±0.00^a^ |
| 10 | Benzaldehyde | 13.264 | - | - | - | - | - | - | - | 0.10±0.01^b^ | 0.10±0.00^b^ | - | - | 0.28±0.00^a^ | 0.25±0.02^c^ | - | 0.37±0.03^a^ | 0.29±0.03^b^ | - | 0.35±0.04^a^ |
| 11 | 4-Ethyl-benzaldehyde | 18.540 | - | - | - | - | - | - | - | - | - | - | - | 0.14±0.01^a^ | - | - | - | - | - | 0.17±0.03^a^ |
| 12 | 2-nonenal | 18.581 | - | - | - | - | - | - | - | - | - | - | - | 0.11±0.00^a^ | - | - | - | 0.16±0.02^a^ | - | 0.15±0.01^a^ |
| 13 | Decenal | 19.291 | - | - | - | - | - | - | - | - | - | - | - | 0.12±0.01^a^ | - | - | - | - | - | - |
| 14 | Pentadecanal | 27.069 | - | - | - | - | - | - | - | - | - | - | - | 0.16±0.01^a^ | - | - | - | - | - | 0.09±0.01^a^ |
| 15 | 4-Pentyl-benzaldehyde | 23.902 | - | - | - | - | - | - | - | - | - | - | - | - | - | - | - | - | - | 0.04±0.00^a^ |
| Aldehydes | | | 2.68±0.11^f^ | 3.44±0.06^d^ | 3.20±0.03^e^ | 9.90±0.10^c^ | 18.72±0.14^b^ | 20.59±0.22^a^ | 2.71±0.12^f^ | 3.23±0.06^e^ | 3.69±0.05^d^ | 10.64±0.62^c^ | 14.82±0.14^b^ | 21.79±0.84^a^ | 8.52±0.30^e^ | 4.55±0.28^f^ | 14.06±0.73^d^ | 21.15±0.97^c^ | 26.60±1.13^b^ | 33.74±6.20^a^ |
| 16 | 3-methyl-1-pentanol | 9.292 | - | - | - | 5.46±0.48^a^ | 4.69±0.95^b^ | - | - | - | - | 3.10±0.27^c^ | 3.34±0.29^b^ | 6.24±0.59^a^ | - | - | 5.17±0.48^a^ | 4.00±0.17^b^ | - | 3.21±0.02^c^ |
| 17 | 1-hexanol | 9.333 | - | - | - | - | - | - | - | - | 0.54±0.04^b^ | 3.65±0.25^a^ | - | - | 1.45±0.04^c^ | - | 1.00±0.02^d^ | 4.07±0.47^a^ | 1.60±0.04^b^ | - |
| 18 | 1-octen-3-ol | 14.016 | 1.24±0.05^c^ | 0.93±0.04^d^ | 1.35±0.11^c^ | 1.81±0.20^b^ | 1.87±0.03^b^ | 2.16±0.10^a^ | 0.64±0.03^f^ | 1.92±0.02^d^ | 1.47±0.09^e^ | 2.59±0.28^b^ | 2.43±0.09^c^ | 8.69±0.51^a^ | 4.49±0.13^f^ | 7.55±0.44^c^ | 5.26±0.31^e^ | 6.49±0.40^d^ | 8.20±0.12^b^ | 11.46±0.35^a^ |
| 19 | 2-ethyl-1-hexanol | 15.492 | - | 0.22±0.01^a^ | - | - | - | - | - | - | - | - | - | 0.30±0.03^a^ | - | - | - | - | - | 0.23±0.01^a^ |
| 20 | 2-propyl-1-pentanol | 15.497 | - | - | 0.30±0.01^a^ | - | - | - | - | - | - | 0.12±0.01^a^ | 0.12±0.01^a^ |  | 0.41±0.05^a^ | - | - | - | 0.21±0.00^b^ | - |
| 21 | Cyclooctanol | 16.507 | 0.21±0.02^c^ | 0.53±0.04^a^ | 0.25±0.02^v^ | 0.35±0.04 | 0.53±0.02^a^ | 0.35±0.01^b^ | - | 0.12±0.00^c^ | - | 0.36±0.04^b^ | 0.31±0.02^b^ | 1.20±0.08^a^ | 0.40±0.07^d^ | - | 0.57±0.09^c^ | 0.70±0.10^b^ | 0.23±0.16^e^ | 0.84±0.07^a^ |
| 22 | 3-Ethyl-4-Nonol | 17.146 | - | - | - | 1.26±0.17^b^ | 2.51±0.07^a^ | 0.74±0.09^c^ | - | - | - | 0.68±0.04^a^ | - | - | - | - | 0.90±0.04^c^ | 1.23±0.04^b^ | 1.91±0.07^a^ | - |
| 23 | 4-methyl-5-decanol | 17.148 | - | - | - | - | - | 0.81±0.03^a^ | - | - | - | - | - | - | - | - | - | - | - | - |
| 24 | 1-Nonanol | 18.823 | - | 0.60±0.01^d^ | 0.21±0.01^e^ | 2.27±0.08^a^ | 0.72±0.10^c^ | 1.08±0.13^b^ | - | - | 0.22±0.01^d^ | 3.28±0.47^a^ | 2.26±0.01^c^ | 2.74±0.19^b^ | - | - | 6.08±0.52^a^ | 5.42±0.47^b^ | 2.36±0.24^c^ | 0.16±0.01^d^ |
| 25 | 3-methyl-1-butanol | 4.049 | - | - | - | - | - | - | - | - | - | - | - | 1.55±0.29^a^ | - | - | - | - | - | - |
| 26 | Phenylethanol | 17.581 | - | - | - | - | - | - | - | - | - | - | - | 0.33±0.02^a^ | - | - | - | - | - | - |
| 27 | 2,3-butanediol | 5.558 | - | - | - | - | - | - | - | 0.04±0.00^a^ | - | - | - | - | - | - | - | - | - | - |
| 28 | 1-Pentanol | 4.927 | - | - | - | - | - | - | - | - | - | - | - | - | 0.21±0.01^b^ | - | - | - | - | 0.64±0.02^a^ |
| 29 | 4-Ethylcyclohexanol | 15.577 | - | - | - | - | - | - | - | - | - | - | - | - | 0.19±0.04b^b^ | - | 0.16±0.01^b^ | 0.13±0.02^c^ | - | 0.31±0.03^a^ |
| 30 | 1-Tetradecanol | 26.619 | - | - | - | - | - | - | - | - | - | - | - | - | - | - | - | 0.18±0.01^a^ | - | - |
| Alcohols | | | 1.45±0.05^f^ | 2.33±0.04^e^ | 2.69±0.11^d^ | 11.15±0.52^a^ | 10.32±0.97^b^ | 5.14±0.13^c^ | 0.64±0.03^f^ | 2.08±0.02^e^ | 2.23±0.05^d^ | 13.78±0.64^b^ | 8.46±0.09^c^ | 21.05±0.98^a^ | 7.15±0.07^f^ | 7.55±0.44^e^ | 19.14±0.85^b^ | 22.22±2.78^a^ | 14.51±0.14^d^ | 16.85±0.15^c^ |
| 31 | Dihydro-5-pentyl-furanone | 22.401 | - | - | - | 0.16±0.00^a^ | - | - | - | - | - | 0.13±0.03^a^ | - | - | - | - | 0.23±0.02^a^ | 0.23±0.04^a^ | 0.13±0.01^c^ | 0.19±0.01^b^ |
| 32 | 2-heptanone | 10.442 | - | - | - | - | - | - | - | - | - | - | - | 0.18±0.00^a^ | - | 0.19±0.01^a^ | - | - | - | - |
| 33 | 3-Octanone | 14.232 | - | - | - | - | - | - | - | - | - | - | - | - | - | - | - | - | 0.57±0.07^a^ | - |
| 34 | 2-Octanone | 14.376 | - | - | - | - | - | - | - | - | - | - | - | - | - | - | - | - | 0.16±0.01^a^ | - |
| Ketones | | | - | - | - | 0.16±0.00^a^ | - | - | - | - | - | 0.13±0.03^b^ | - | 0.18±0.00^a^ | - | 0.19±0.01^c^ | 0.23±0.02^b^ | 0.23±0.04^b^ | 0.86±0.02^a^ | 0.19±0.01^c^ |
| 35 | Tetradecane | 22.920 | - | 0.04±0.00^a^ | - | - | - | - | 0.19±0.01^a^ | - | - | 0.12±0.00^b^ | - | - | - | - | - | 0.04±0.00^b^ | - | 0.10±0.01^a^ |
| 36 | Hexadecane | 25.675 | 0.26±0.01^a^ | - | 0.14±0.01^c^ | 0.19±0.06^b^ | - | 0.30±0.01^a^ | 0.16±0.01^a^ | 0.11±0.02^b^ | 0.17±0.01^a^ | 0.17±0.02^a^ | 0.17±0.00^a^ | 0.20±0.00^a^ | 0.03±0.00^c^ | 0.14±0.00^a^ | 0.08±0.00^b^ | 0.08±0.01^b^ | - | 0.09±0.00^b^ |
| 37 | Heptadecane | 26.872 | 0.26±0.01^a^ | 0.16±0.00^b^ | 0.18±0.00^b^ | 0.24±0.04^a^ | - | 0.54±0.03 | 0.16±0.00^a^ | 0.17±0.00^a^ | - | 0.18±0.01^a^ | 0.14±0.00^a^ | 0.19±0.04^a^ | - | 0.15±0.00^a^ | 0.08±0.00^b^ | 0.11±0.05^a^ | 0.08±0.02^b^ | 0.09±0.01^b^ |
| 38 | Heneicosane | 27.021 | 0.19±0.01^c^ | - | 0.14±0.03^c^ | 0.28±0.05^b^ | - | 0.59±0.05^a^ | 0.21±0.00^a^ | - | - | - | - | 0.25±0.02^a^ | - | - | 0.04±0.00^a^ | - | - | - |
| 39 | Octadecane | 27.99 | - | - | 0.12±0.02^b^ | 0.30±0.00^a^ | - | 0.30±0.03^a^ | - | 0.15±0.01^b^ | 0.37±0.03^a^ | 0.18±0.01^b^ | - | 0.20±0.03^b^ | - | - | 0.09±0.01^c^ | 0.13±0.03^b^ | - | 0.19±0.01^a^ |
| 40 | Nonadecane | 29.04 | - | - | - | 0.23±0.01^b^ | - | 0.59±0.11^a^ | 0.15±0.01^a^ | - | - | 0.14±0.00^a^ | - | - | - | - | - | 0.08±0.00^b^ | - | 0.26±0.02^a^ |
| 41 | Tridecane | 21.268 | - | - | - | - | - | - | - | - | - | - | - | 0.09±0.01^a^ | - | - | - | - | - | - |
| Hydrocarbons | | | 0.71±0.01^c^ | 0.20±0.00^e^ | 0.58±0.01^d^ | 1.24±1.06^b^ | - | 2.32±0.01-3^a^ | 0.87±0.01^b^ | 0.43±0.01^d^ | 0.54±0.01^d^ | 0.79±0.02^c^ | 0.31±0.00^e^ | 1.03±0.04^a^ | 0.03±0.00^e^ | 0.29±0.00^c^ | 0.29±0.01^c^ | 0.44±0.01^b^ | 0.08±0.02^d^ | 0.73±0.01^a^ |
| 42 | Ethyl Formate | 13.731 | - | - | - | - | 1.10±0.05^a^ | - | - | - | - | - | - | - | - | - | - | - | - | - |
| 43 | Heptyl formate | 13.745 | - | 0.77±0.33^b^ | - | 1.05±0.07^a^ | - | 0.42±0.05^c^ | - | - | - | 1.28±0.27^a^ | 0.86±0.01c^b^ | 1.30±0.12^a^ | 2.90±0.29^a^ | - | 1.37±0.03^b^ | 1.29±0.03^c^ | 0.59±0.04^d^ | 0.35±0.02^e^ |
| 44 | Ethylene hexanoate | 14.184 | 0.14±0.01^b^ | 0.25±0.02^a^ | - | - | - | - | - | - | - | 0.43±0.02^a^ | 0.47±0.11^a^ | - | 1.32±0.04^b^ | - | - | 1.43±0.13^a^ | - | - |
| 45 | Octyl formate | 16.576 | 0.48±0.02^e^ | 1.31±0.02^c^ | 0.80±0.02^d^ | 3.82±0.36^a^ | 3.12±0.15^b^ | 3.01±0.16^b^ | 1.71±0.10^d^ | 0.31±0.02^f^ | 0.74±0.02^e^ | 3.14±0.22^b^ | 2.92±0.18^c^ | 3.40±0.15^a^ | 0.69±0.05c ^a^ | - | - | 3.08±0.12^a^ | 1.10±0.14^b^ | 0.66±0.05^c^ |
| 46 | Diethyl succinate | 19.026 | - | 0.33±0.01^a^ | - | - | - | - | - | - | - | - | - | - | - | - | 0.10±0.01^a^ | - | - | - |
| 47 | Hept-4-ylisobutyl phthalate | 28.798 | - | 0.42±0.02^c^ | - | 0.73±0.08^b^ | 0.18±0.01^d^ | 2.39±0.17^a^ | 0.28±0.01^a^ | 0.29±0.01^a^ | 0.06±0.00^b^ | 0.33±0.01^a^ | 0.32±0.07^a^ | - | - | 0.11±0.01^a^ | - | 0.12±0.01^a^ | - | - |
| 48 | Dibutyl phthalate | 29.755 | 0.28±0.01^d^ | 0.15±0.01^e^ | - | 0.75±0.08^b^ | 0.49±0.02^c^ | 2.93±0.05^a^ | 0.40±0.07^a^ | 0.44±0.01^a^ | 0.35±0.01b ^a^ | 0.15±0.00^d^ | 0.25±0.03^c^ | - | 0.15±0.00^a^ | 0.14±0.01^a^ | - | - | - | 0.20±0.01^a^ |
| 49 | Diethylaminodithioformate methyl ester | 22.74 | - | - | - | - | - | - | 2.15±0.11^b^ | 2.74±0.01^a^ | 1.84±0.07^c^ | - | - | 0.53±0.03^d^ | 0.73±0.10^c^ | 1.43±0.16^a^ | 0.95±0.07^b^ | 0.25±0.04^e^ | 0.37±0.01^d^ | 0.14±0.01^f^ |
| 50 | 2-methyl-2-butyric acid methyl ester | 9.156 | - | - | - | - | - | - | - | - | - | - | - | - | - | 0.56±0.05^a^ | - | - | - | - |
| 51 | Ethyl octanoate | 19.350 | - | - | - | - | - | - | - | - | - | - | - | - | - | - | - | - | - | 0.38±0.02^a^ |
| 52 | Ethyl decanoate | 22.86 | - | - | - | - | - | - | - | - | - | - | - | - | - | - | - | - | - | 0.36±0.03^a^ |
| 53 | Ethyl laurate | 25.614 | - | - | - | - | - | - | - | - | - | - | - | - | - | - | - | - | - | 0.11±0.00^a^ |
| 54 | Hexadecanoic acid ethyl ester | 29.992 | - | - | - | - | - | - | - | - | - | - | - | - | - | - | - | 0.57±0.11^a^ | - | - |
| Esters | | | 0.90±0.01^e^ | 3.23±0.33^d^ | 0.80±0.02^e^ | 6.35±0.21^b^ | 4.89±0.07^c^ | 8.75±0.10^a^ | 4.54±0.12^d^ | 3.78±0.02^e^ | 2.99±0.03^f^ | 5.33±0.32^a^ | 4.82±0.06^c^ | 5.23±0.14^b^ | 5.79±0.30^a^ | 2.24±0.04^c^ | 2.42±0.04^b^ | 6.74±0.08^a^ | 2.06±0.06^d^ | 2.20±0.02^c^ |
| 55 | Methoxyphenoxime | 11.760 | 0.99±0.10^c^ | 1.51±0.04^b^ | 0.82±0.10^d^ | 0.87±0.06^c^ | 1.86±0.06^a^ | - | 0.83±0.09^d^ | 0.97±0.07^c^ | 2.83±0.04^a^ | 0.56±0.08^e^ | 0.82±0.09^d^ | 1.26±0.09^b^ | 0.54±0.04^e^ | 1.08±0.14^b^ | 0.83±0.12^c^ | 0.71±0.07^d^ | 5.44±0.48^a^ | 1.08±0.06^b^ |
| 56 | 2,4-di-tert-butylphenol | 24.581 | 0.43±0.05^c^ | - | - | - | 0.52±0.02^b^ | 0.76±0.04^a^ | 0.18±0.05b ^a^ | 0.18±0.01^b^ | - | 0.33±0.01^a^ | - | - | 0.07±0.00^a^ | - | - | - | - | - |
| 57 | Butylated hydroxytoluene | 24.642 | 0.10±0.00^a^ | 0.12±0.01^a^ | 0.11±0.00^a^ | 0.11±0.00^a^ | 0.11±0.00^a^ | 0.12±0.01^a^ | 0.14±0.01^a^ | 0.08±0.00^a^ | 0.09±0.00^a^ | 0.13±0.01^a^ | 0.12±0.00^a^ | 0.09±0.01^a^ | 0.15±0.01^d^ | 0.91±0.08^a^ | 0.37±0.01^b^ | 0.19±0.01^d^ | 0.25±0.01^c^ | 0.33±0.05^b^ |
| 58 | Diphenylamine | 26.067 | - | - | - | - | - | - | 0.17±0.02^a^ | 0.18±0.04^a^ | - | - | - | - | - | 0.13±0.01^a^ | - | - | - | - |
| Benzodiazepines | | | 1.52±0.05^b^ | 1.63±0.04b ^a^ | 0.93±0.10^c^ | 0.98±0.06^c^ | 2.49±0.06^a^ | 0.88±0.04^d^ | 1.32±0.10^c^ | 1.41±0.07^b^ | 2.92±0.04^a^ | 1.02±0.08^e^ | 0.94±0.09^f^ | 1.35±0.09^d^ | 0.76±0.04^f^ | 2.12±0.15^b^ | 1.20±0.12^d^ | 0.90±0.07^e^ | 5.69±0.48^a^ | 1.41±0.06^c^ |
| 59 | 2-methylvaleric acid | 14.187 | - | - | - | - | - | - | - | - | - | 0.45±0.04^b^ |  | 2.41±0.12^a^ | - | - | 1.53±0.08^b^ | - | - | 2.23±0.14^a^ |
| 60 | Acetic acid | 4.608 | - | - | - | - | - | - | - | - | - | - | - | - | - | - | - | - | 0.23±0.00^a^ | - |
|  | Acids |  | - | - | - | - | - | - | - | - | - | 0.45±0.04^b^ | - | 2.41±0.12^a^ | - | - | 1.53±0.08^b^ | - | 0.23±0.00^c^ | 2.23±0.14^a^ |
| 61 | 2- (1,1-dimethylethyl) -3-methyloxypyran | 4.068 | - | - | - | - | - | - | - | - | - | - | - | 1.44±0.03^a^ | - | 2.29±0.09^a^ | 0.39±0.07^c^ | - | - | 0.95±0.11^b^ |
| 62 | 2-pentyl-furan | 14.360 | - | - | - | - | - | - | - | - | - | - | - | 0.81±0.02^a^ | - | - | 0.74±0.53^a^ | 0.40±0.07^b^ | - | 0.46±0.04^b^ |
| 63 | Indole | 21.239 | - | - | - | - | - | - | - | - | - | - | - | - | - | 0.32±0.03^b^ | 0.39±0.03^a^ | - | - | - |
| Heterocycles | | | - | - | - | - | - | - | - | - | - | - | - | 2.25±0.06^a^ | - | 2.61±0.07^a^ | 1.52±0.42^b^ | 0.40±0.07^d^ | - | 1.41±0.11^c^ |
| 64 | Dimethyl tetrasulfide | 19.77 | - | - | - | - | - | - | - | - | - | - | - | - | 0.52±0.10^a^ | - | - | - | - | - |
| 65 | N, N-dibutyl-formamide | 21.378 | - | - | - | - | - | - | - | - | - | - | - | - | 0.32±0.01^c^ | 0.39±0.03^b^ | 0.80±0.12^a^ | - | - | - |
| 66 | Hexadecyl epoxyethane | 25.857 | - | - | - | - | - | - | - | - | - | - | - | - | - | - | - | - | - | 0.04±0.00^a^ |
| Other | | | - | - | - | - | - | - | - | - | - | - | - | - | 0.84±0.02^a^ | 0.39±0.03^b^ | 0.80±0.12^a^ | - | - | 0.04±0.00^c^ |

“-”, Not detected.

The different capital letters indicate statistically significant differences between different treatments during storage (*P* < 0.05).
